# Digital Mental Health Treatment and Symptoms of Depression and Anxiety in Breast Cancer Survivors: A Randomized Clinical Trial

**DOI:** 10.1001/jamanetworkopen.2026.23871

**Published:** 2026-07-20

**Authors:** Philip I. Chow, Wen You, Kelly M. Shaffer, Wendy F. Cohn, Shayna L. Showalter, Cynthia Orange, Lee M. Ritterband, David C. Mohr

**Affiliations:** 1 Center for Behavioral Health and Technology, Department of Psychiatry and Neurobehavioral Sciences, University of Virginia School of Medicine, Charlottesville; 2 University of Virginia Comprehensive Cancer Center, Charlottesville; 3 Center for Behavioral Intervention Technologies, Department of Preventive Medicine, Northwestern University Feinberg School of Medicine, Chicago, Illinois

## Abstract

**Importance:**

Psychological distress in breast cancer survivors (BCS) is highly prevalent. Access to treatment is limited by barriers, including a lack of psychotherapists.

**Objective:**

To evaluate the efficacy of a scalable, publicly accessible digital mental health treatment (DMHT) for reducing symptoms of depression and anxiety in BCS and the effect of human coaching on engagement among BCS with initially low engagement.

**Design, Settings, and Participants:**

This randomized clinical trial followed the SMART (Sequential, Multiple Assignment, Randomized Trial) design and was conducted in the US between September 2021 and January 2024. Adult women who were within 5 years of receiving a breast cancer diagnosis (stage I-III), had a smartphone with a data plan, and had elevated symptoms of depression or anxiety were eligible for inclusion. Participants completed self-reported questionnaires at baseline, 8-week postintervention period, and 6-month and 12-month follow-up periods. Participants were randomized 2:1 to either the DMHT (IntelliCare) or the patient education program. Data analysis was based on intention-to-treat principle.

**Interventions:**

The DMHT program is an 8-week self-guided, fully automated suite of 5 clinically focused applications. Participants in the DMHT group were classified as having optimal or suboptimal engagement based on week 1 use. Those with suboptimal engagement were subsequently randomized 1:1 to receive additional coaching or no coaching. The patient education program is an active control condition consisting of 8 weeks of use of a psychoeducational application and no human support.

**Main Outcomes and Measures:**

Primary outcomes were symptoms of anxiety (self-assessed using the 7-item Generalized Anxiety Disorder) and depression (self-assessed using the 8-item Patient Health Questionnaire). Secondary outcomes were number of days of application use and number of application sessions.

**Results:**

A total of 313 women (mean [SD] age, 51.62 [10.51] years) were enrolled and assigned to the DMHT program (n = 209) or the patient education program (n = 104). Primary analyses included 272 participants. Participants in both DMHT and patient education programs showed significantly reduced symptoms of both depression (Cohen *d* = −0.60 at the 8-week, −1.03 at the 6-month, and −1.13 at the 12-month assessment) and anxiety (Cohen *d* = −0.68 at the 8-week, −0.80 at the 6-month, and −0.85 at the 12-month assessment) from baseline. Compared with participants in the patient education group, those in the DMHT group showed significantly greater reductions in symptoms of anxiety (average marginal effect [AME] = −1.43; *z* = −2.65 [95% CI, −2.49 to −0.38]; *P* = .01) but not for symptoms of depression (AME = −0.98; *z* = −1.89 [95% CI, −1.99 to 0.04]; *P* = .06). Compared with participants with suboptimal engagement who did not receive coaching, those who received coaching used the intervention for a significantly greater number of days (mean [SD], 27.48 [0.94] vs 24.04 [1.16] days; *P* = .02).

**Conclusions and Relevance:**

This randomized trial found that BCS can benefit from a DMHT program, particularly for reducing anxiety. Coaching may promote consistent engagement with a DMHT for those with difficulty engaging initially.

**Trial Registration:**

ClinicalTrials.gov Identifier: NCT04583891

## Key Points

**Question:**

Can a digital mental health treatment (DMHT) mitigate symptoms of anxiety and depression in breast cancer survivors with elevated symptoms?

**Findings:**

In this randomized clinical trial of 313 women with breast cancer, compared with participants who received a smartphone-delivered psychoeducation program, those assigned to the smartphone-delivered DMHT program reported significantly greater reductions in symptoms of anxiety but not depression. Among participants who showed suboptimal DMHT engagement during the first intervention week, human coaching (vs no coaching) increased the number of days of application use.

**Meaning:**

The findings of this trial indicate that breast cancer survivors with symptoms of anxiety may benefit from a scalable and accessible DMHT program.

## Teaser

This randomized clinical trial examines the efficacy of a self-guided suite of mental health–focused applications with as-needed human support to reduce symptoms of anxiety and depression among women with breast cancer.

## Introduction

Nearly half of all women diagnosed with invasive breast cancer report clinically significant symptoms of depression or anxiety within the first year of their diagnosis.^1,2,3,4^ For many of these individuals, the symptoms persist up to 5 years after diagnosis^1^ and are associated with poor quality of life,^5^ increased mortality,^6,7^ and high economic costs.^8^ As the breast cancer survival rate continues to increase,^9^ so too does the number of breast cancer survivors (BCS) who require mental health support.^10^

Interventions that emphasize skills acquisition, such as cognitive behavioral therapy, effectively reduce symptoms of depression and anxiety in BCS.^11,12,13,14^ Yet, access to evidence-based mental health treatments remains limited due to numerous obstacles,^15,16^ including a severe shortage of psychotherapists.^17,18,19^ As a result, nearly half of BCS report unmet psychosocial needs.^20,21,22,23^ Digital mental health treatments (DMHTs) delivered through smartphone applications may help overcome these obstacles.^24,25,26,27^ In addition to being highly scalable, DMHTs reduce many barriers associated with in-person care because treatment is affordable, readily available, and not limited by proximity to psychotherapists.

The IntelliCare program is one such DMHT, designed to promote skills acquisition through brief interactions targeting specific clinical concerns (eg, worry and behavioral inhibition).^28,29,30,31,32^ Built to fit into daily life, this program has strong dissemination potential for the large population of distressed BCS in the US.^29^ Numerous studies demonstrate the efficacy of DMHTs for reducing symptoms of depression and anxiety in the general population.^28,33,34,35,36^

While DMHTs are poised to improve access to high-quality treatment, sustained engagement is a common problem. Engagement is critical given the typical dose-response relationship repeatedly observed both in psychological treatment broadly^37^ and in digital health interventions specifically.^38,39^ Some digital interventions have provided all users with remote coaching via telephone calls and text messaging.^28,40,41^ However, universally providing coaching may be unnecessary and cost-prohibitive for public dissemination. One promising approach is to use an adaptive strategy that provides coaching only to users who cannot initially engage.^42,43,44^ Before such a strategy can be implemented, the incremental benefit of providing coaching to those with early engagement difficulty must be examined.^43,44^

In this randomized clinical trial (RCT), we aimed to evaluate the efficacy of a scalable, publicly accessible DMHT for reducing symptoms of depression and anxiety in BCS and the effect of human coaching on engagement among BCS with low engagement in the first week of the intervention. We hypothesized that the DMHT (primary intervention program) would produce greater reductions in depression and anxiety symptoms than smartphone-delivered patient education (control intervention program) across the postintervention and follow-up assessment periods. Additionally, evaluating coaching designed to address barriers to DMHT use^41,45^ would help improve engagement among BCS with suboptimal early engagement.

## Methods

### Design

This study followed the Sequential, Multiple Assignment, Randomized Trial (SMART)^44^ design and recruited participants across the US between September 2021 and November 2022. Final data collection occurred in January 2024. The University of Virginia Institutional Review Board for Health Sciences Research approved this RCT. Supplement 1 provides the trial protocol. Digital informed consent was obtained from all participants. We followed the Consolidated Standards of Reporting Trials (CONSORT) reporting guideline.^46^

### Participants

Eligible participants were women 18 years or older who were diagnosed with breast cancer (stages I-III) in the previous 5 years, had a smartphone with a data plan, and were screened with high symptoms of depression (score of ≥10 on the 8-item Patient Health Questionnaire [PHQ-8]^47^) or anxiety (score of ≥8 on the 7-item Generalized Anxiety Disorder [GAD-7]^48^). Exclusion criteria included a severe mental health condition (psychosis, bipolar disorder, or active suicidal ideation) to avoid interference with study procedures, current psychotherapy for depression or anxiety to avoid treatment interference, or taking psychotropic medication that was not stable over the past 2 weeks to avoid medication placebo effects.

### Procedure

Targeted nationwide social media advertising was supplemented by advertising in University of Virginia Cancer Center clinics and the surrounding catchment area. Recruited individuals completed an online screener, followed by a telephone interview. All participants provided informed consent through a digital signature on an online form after telephone discussion with study staff. Participants completed a baseline assessment before first randomization, an 8-week postintervention assessment, and 6-month and 12-month follow-up assessments using a Health Insurance Portability and Accountability Act–compliant online survey tool (REDCap). The principal investigator (P.I.C.) and statistician (W.Y.) were blind to participants’ conditions during the trial. Given the nature of the intervention programs, we were unable to blind participants to their condition.

### Randomization and Onboarding

Following the baseline assessment, participants were randomized 2:1 to receive the DMHT or the patient education program, respectively, for 8 weeks (Figure). An independent statistician created a computer-generated randomization sequence with a 2 to 1 ratio in randomly permutated blocks of 12 and 9. Randomization assignment was concealed until after entry criteria were confirmed. Participants were instructed by email to download the DMHT application (IntelliCare Hub)^28^ or the patient education application (Mood Education) on their smartphone, with the option of calling study staff to help guide the application downloading process. Participants randomized to receive the DMHT were emailed information about its applications and a suggestion to try a new application every week, consistent with prior program trials.^28,49^

**Figure.**
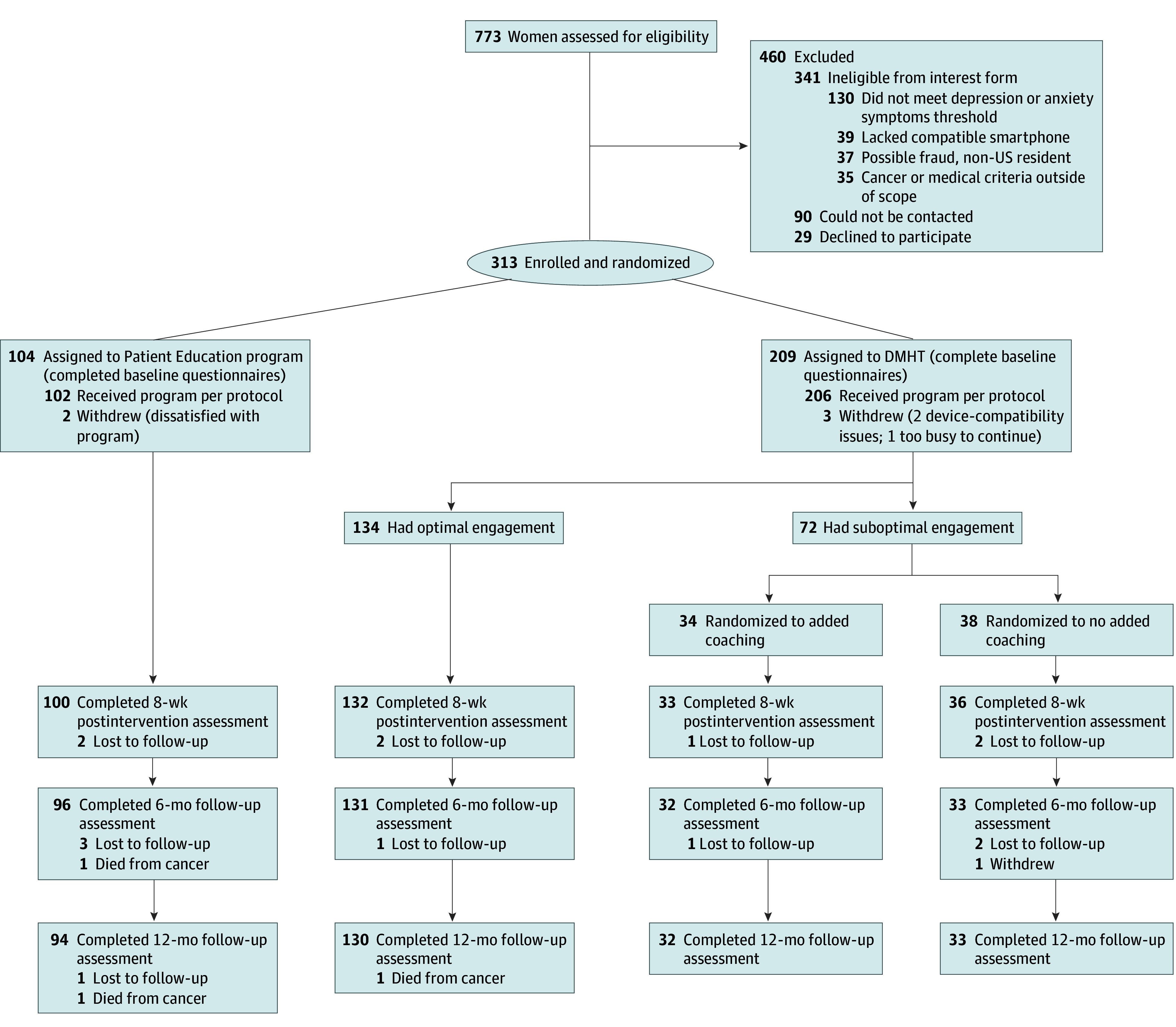
Study Flow Diagram DMHT indicates digital mental health treatment.

Daily DMHT use was monitored during week 1. Participants were classified as having optimal or suboptimal engagement based on the ideal use case for the Day to Day application, which delivers daily prompts to open the application. Those who launched the application on 6 or more days in week 1 were categorized as having optimal engagement and continued without additional support. Those who launched the application on fewer than 6 days were categorized as having suboptimal engagement and were again randomized 1:1 (using a computer-generated randomization sequence with a 1 to 1 ratio in randomly permutated blocks of 6 and 8) to receive additional coaching starting on week 2 or no additional coaching (Figure).

### Intervention Conditions

#### DMHT

The DMHT program is an 8-week self-guided, fully automated suite of 5 clinically focused applications (Table 1) designed to promote skills acquisition through brief check-ins.^28,29,30,31,32^ A web-based dashboard allowed research staff to monitor participant activity on each application. A Hub application integrates the experience for the user, organizes notifications, and provides information about each of the 5 applications. A back-end system reminds users to download and try a clinical application every week, starting with the Day to Day application for week 1. Participants were encouraged to try the recommended application each week but could download and use any application at any time. When participants used each application, they were instructed to continue mastering skills within the application that they found most helpful.

**Table 1.** Overview of the Digital Mental Health Treatment Program

Application name	Psychological strategy	Function
Day to Day	Psychoeducation	Daily tidbits of knowledge to bolster mood
Daily Feats	Goal setting	In-application calendar to track successes and identified tasks to complete
Thought Challenger	Thought restructuring	Cognitive restructuring exercises to challenge unhelpful thoughts
My Mantra	Positive affirmations	Generation of personal mantras and photo albums as reminders
Worry Knot	Emotion regulation	Interactive exercises to decrease worry
Hub	Reminders	Management of reminders, messages, and downloads for all 5 applications

#### Coaching (DMHT Only)

Coaching started in week 2 and continued through the end of the intervention (week 8). Based on the Efficiency Model of Support,^41^ coaching supports participants’ use of DMHTs following a manualized procedure. Coaches work with participants to identify and address barriers to benefitting from the program.^25^ Coaches are explicitly instructed not to provide counseling or psychotherapy.^28,50^

Two coaches—one with a bachelor’s degree and one with a master’s degree—received training in the coaching manual^51^ and weekly supervision. Participants received a coaching welcome packet by email, an onboarding telephone call (approximately 30-45 minutes), and the option for a midtreatment call (approximately 10-15 minutes) at week 4. Additional communication was managed via text messaging. Coaches typically sent text messages every week to encourage and remind participants to use DMHT regularly. Participants could contact coaches via text messaging with program-related questions.

#### Patient Education 

The patient education program is an active control condition consisting of 8 weeks of a psychoeducational application.^52^ This application contained static evidence-based and extensive content on depression and anxiety symptoms, causes, and prevalence as well as instruction in evidence-based techniques, such as diaphragmatic breathing. Participants were instructed to use the patient education application as often as they desired. They did not receive notifications to use the application nor did they receive human support.

### Outcomes

Primary outcomes were symptoms of depression (self-assessed using PHQ-8; score range: 0-24, with the highest score indicating the greatest depression symptom severity) and anxiety (self-assessed using GAD-7; score range: 0-21, with the highest score indicating the greatest anxiety symptom severity). These symptoms were assessed at baseline, 8-week postintervention, and 6-month and 12-month follow-up periods.

Secondary outcomes were number of days of application use and number of application sessions. Data related to these application engagement metrics were captured throughout the active study period (weeks 1-8). In keeping with prior DMHT program trials, an application session is defined as a user-initiated event after 5 minutes of no activity.^28^

### Statistical Analysis

The target analytical sample size (n = 250) was based on the main contrast between the DMHT and patient education programs, to determine the overall marginal effect of initial treatment assignment. Power calculations assumed a medium effect size for depression or anxiety symptoms (Cohen *d* = 0.35) based on a 2-sided test with 80% power and 5% type I error rate. To account for an anticipated 20% attrition rate across follow-up assessments, the target recruitment sample size was 313.

Depression and anxiety symptoms were analyzed using extended generalized estimating equations (GEEs), as recommended for SMART study designs,^53^ to model repeated measures collected at each assessment time point. GEEs use all available observations under the missing-at-random assumption and provide valid population-mean inferences without requiring imputation. To account for the sequential randomization in a SMART study, inverse probability weights were computed based on participants’ randomization probabilities to obtain unbiased estimates of marginal treatment effects (MTEs). Models included main effects for treatment group and time as well as their interaction. An exchangeable working correlation structure was specified to account for within-participant dependence, and robust SEs were used to accommodate variability introduced by weighting and potential correlation misspecification. All models were adjusted for covariates specified a priori, including age, race (Asian, Black or African American, White, and other [including American Indian or Alaska Native, Native Hawaiian or Other Pacific Islander, or multiracial]), and cancer stage. Self-reported race was collected from participants in this study to promote transparency of findings and to evaluate generalizability of findings to the broader population of breast cancer survivors.

Within-group effect sizes (Cohen *d*) contextualize symptom changes over time and were calculated as the adjusted mean change from baseline to each assessment period, standardized by the baseline SD and weighted to account for differences in group sample sizes. To estimate the effect of added coaching on the secondary outcomes of engagement, a probability-weighted GEE^54^ was used to test whether participants with suboptimal engagement who received (vs did not receive) coaching after week 1 engaged more with the application during the remainder of the intervention period. For all statistical tests, *P* < .05 was considered statistically significant. Additional exploratory analyses and results comparing the 2 embedded dynamic treatment regimens of the DMHT program and the impact of coaching on primary outcomes for suboptimal engagement can be found in the eAppendix in Supplement 2. Data analysis was based on intention-to-treat principle and was performed using Stata, version 18 (StataCorp LLC).

## Results

Of the 773 women screened, 313 enrolled (40.5%) across 44 US states, of whom 209 were randomly assigned to receive the DMHT program and 104 were randomly assigned to the patient education program. Among participants, the mean (SD) age was 51.62 (10.51) years, and 4 (1.3%) self-reported as Asian; 25 (8.0%) as Black or African American; 20 (6.5%) as Hispanic or Latina; 288 (93.5%) as non–Hispanic or Latina; and 273 (87.5%) as White individuals, while 10 (3.2%) identified as other race and ethnicity. Most participants reported being employed (187 [64.7%]), being married (221 [70.6%]), completing a college degree (269 [86.2%]), and having Stage I breast cancer (175 [56.3%]). At baseline, 239 participants (76.4%) reported elevated depression symptoms, 278 (88.8%) reported elevated anxiety symptoms, and 204 (65.2%) met the criteria for both depression and anxiety. Sample characteristics are presented in Table 2. The lost-to-follow-up rate was low, with 301 participants (96.2%) completing the 8-week postintervention assessment, 292 (93.3%) completing the 6-month follow-up assessment, and 289 (92.3%) completing the 12-month follow-up assessment (Figure).

**Table 2.** Descriptive Characteristics of the Full Sample at Baseline

Variable	Women, No. (%)		
	Total sample (N = 313)	DMHT (n = 209)	Patient education (n = 104)
Descriptive			
Age, mean (SD), y	51.62 (10.51)	51.36 (10.68)	52.17 (10.18)
Race^a^			
Asian	4 (1.3)	4 (1.9)	0
Black or African American	25 (8.0)	18 (8.6)	7 (6.8)
White	273 (87.5)	182 (87.1)	91 (88.4)
Other^b^	10 (3.2)	5 (2.4)	5 (4.9)
Ethnicity^a^			
Hispanic or Latina	20 (6.5)	15 (7.3)	5 (5.0)
Non–Hispanic or Latina	288 (93.5)	192 (92.8)	96 (95.1)
Educational level			
High school diploma or GED	43 (13.8)	26 (12.5)	17 (16.4)
Associate or Bachelor’s degree	159 (51.0)	106 (51.0)	53 (51.0)
Graduate degree	110 (35.3)	76 (36.5)	34 (32.7)
Employment status			
Yes	187 (64.7)	127 (64.8)	60 (64.5)
No	102 (35.3)	69 (35.2)	33 (35.5)
Marital status			
Married	221 (70.6)	142 (67.9)	79 (76.0)
Separated or divorced	59 (18.9)	42 (20.1)	17 (16.4)
Widowed	8 (2.6)	8 (3.8)	0
Never married	25 (8.0)	17 (8.1)	8 (7.7)
Cancer stage			
1	175 (56.3)	119 (56.9)	56 (54.9)
2	100 (32.2)	71 (34.0)	29 (28.4)
3	36 (11.6)	19 (9.1)	17 (16.7)

Abbreviations: DMHT, digital mental health treatment; GED, General Educational Development.

^a^ Race and ethnicity were self-reported.

^b^ Other includes American Indian or Alaska Native, Native Hawaiian or Other Pacific Islander, and multiracial.

The sample size for the primary analyses included 272 participants. Per the GEE and weighting analyses, those in the DMHT group who received additional coaching (n = 34) were not included in the primary analyses. Three participants in the DMHT group were excluded from the primary analyses because they never received the program, and an additional 4 participants were excluded due to missing covariate data (age, race, or cancer stage). No adverse events were reported.

### Primary Outcomes 

Unadjusted means and SDs of depression and anxiety symptoms at each assessment are presented in Table 3. Primary outcomes analyses indicated that, averaged across the postintervention and follow-up assessments and controlling for the prespecified covariates, BCS in the DMHT group did not report statistically significantly reduced depression symptoms compared with those in the patient education group (average marginal effect [AME] = −0.98; *z* = −1.89 [95% CI, −1.99 to 0.04]; *P* = .06). BCS in the DMHT group reported significantly less anxiety symptoms than those in the patient education group (AME = −1.43; *z* = −2.65 [95% CI, −2.49 to −0.38]; *P* = .01). Time by group interactions indicated no group differences in depression symptoms at any of the 8-week (marginal treatment effect [MTE] [SE] = 0.03 [0.65]; *P* = .97), 6-month (MTE [SE] = −0.92 [0.66]; *P* = .17), and 12-month (MTE [SE] = −0.79 [0.67]; *P* = .24) assessments. No group differences were observed for anxiety symptoms at the 8-week (MTE [SE] = −0.96 [0.67]; *P* = .15), 6-month (MTE [SE] = −0.72 [0.67]; *P* = .28), and 12-month (MTE [SE] = −0.98 [0.70]; *P* = .16) assessments.

**Table 3.** Means of Outcome Measures

Condition	Unadjusted mean scores (SD)			
	Baseline	8-wk Postintervention (n = 301)	6-mo Follow-up (n = 292)	12-mo Follow-up (n = 289)
**PHQ-8: Depression symptoms**				
DMHT				
Optimal engagement	12.13 (4.64)	8.06 (5.10)	7.16 (4.92)	6.71 (4.93)
Suboptimal engagement				
Coached	12.47 (4.46)	7.22 (4.39)	7.19 (5.08)	6.58 (5.58)
Uncoached	11.84 (4.15)	10.50 (5.84)	7.44 (4.34)	7.41 (3.87)
Patient Education	12.69 (4.44)	9.43 (4.92)	8.81 (5.53)	8.34 (5.92)
**GAD-7: Anxiety symptoms**				
DMHT				
Optimal engagement	11.43 (4.05)	6.85 (4.63)	6.26 (4.97)	5.72 (5.11)
Suboptimal engagement				
Coached	12.35 (5.16)	7.69 (5.14)	7.33 (5.67)	7.22 (5.57)
Uncoached	12.24 (4.99)	7.61 (4.48)	7.03 (4.60)	6.00 (4.24)
Patient Education	12.61 (4.68)	8.87 (5.49)	8.01 (5.53)	7.51 (5.75)

Abbreviations: DMHT, digital mental health treatment; GAD-7, 7-item Generalized Anxiety Disorder; PHQ-8, 8-item Patient Health Questionnaire.

Within-group adjusted changes in depression symptoms from baseline were statistically significant at all follow-up assessments for both groups and increased in magnitude over time. For the DMHT group, corresponding Cohen *d* values were −0.60 at the 8-week, −1.03 at the 6-month, and −1.13 at the 12-month assessment. For the patient education group, Cohen *d* values were −0.68 at the 8-week, −0.80 at the 6-month, and −0.85 at the 12-month assessment.

Within-group changes in anxiety symptoms revealed statistically significant reductions from baseline at all assessment time points for both groups. For the DMHT group, Cohen *d* values were −0.95, −1.08, and −1.24 at the 8-week, 6-month, and 12-month assessment, respectively. The corresponding Cohen *d* values for the patient education group were −0.72 at the 8-week, −0.88 at the 6-month, and −0.95 at the 12-month assessment.

### Secondary Outcomes 

As shown in the Figure, participants in the DMHT group with optimal engagement (n = 134) outnumbered those with suboptimal engagement (n = 72) in a ratio of 1.86 to 1. Engagement metrics for all groups are summarized in Table 4. Among those with suboptimal engagement, participants who received additional coaching after week 1 used DMHT applications for significantly greater number of days during the remainder of the intervention period compared with participants who did not receive coaching (mean [SD], 27.48 [0.94] vs 24.04 [1.16] days; *P* = .02). Participants with suboptimal engagement who received additional coaching also had a greater number of sessions after week 1 compared with those who did not have coaching (mean [SD], 46.91 [2.73] vs 40.26 [2.91] sessions; *P* = .10), but this result was not statistically significant.

**Table 4.** Intervention Sessions and Days of Intervention Use by Week

Week of intervention	DMHT, median (IQR)								Patient Education, median (IQR)	
	Optimal engagement		Suboptimal engagement				Overall			
			Additional coaching		No additional coaching					
	Sessions	Days	Sessions	Days	Sessions	Days	Sessions	Days	Sessions	Days
1	12 (10-17)	6 (5-7)	7 (5-9)	4 (3-5)	6 (4-9)	4 (3-5)	10 (7-14)	6 (4-7)	2 (2-4)	2 (1-2)
2	9 (6-14)	6 (4-7)	4.5 (3-8)	3 (2-5)	4 (2-6)	3 (2-4)	8 (4-11)	5 (3-6)	0 (0-1)	0 (0-1)
3	8 (5-12)	5 (4-7)	5 (4-9)	4 (2-5)	2.5 (1-4)	2 (1-4)	7 (3-10)	5 (2-6)	0 (0-0)	0 (0-0)
4	7 (4-11)	5 (3-6)	5 (2-8)	3.5 (2-5)	1 (1-5)	1 (1-4)	6 (3-9)	4 (2-6)	0 (0-0)	0 (0-0)
5	6 (3-9)	5 (3-6)	3.5 (2-6)	3 (2-4)	2 (0-3)	1.5 (0-3)	5 (2-8)	4 (2-5)	0 (0-0)	0 (0-0)
6	5 (2-7)	4 (2-6)	4 (2-6)	3 (2-4)	1 (0-3)	1 (0-3)	4 (1-7)	3 (1-5)	0 (0-0)	0 (0-0)
7	5 (2-8)	4 (1-6)	4.5 (3-7)	4 (3-5)	0.5 (0-4)	0.5 (0-4)	4 (1-7)	3 (1-5)	0 (0-0)	0 (0-0)
8	4 (1-8)	3 (1-5)	3.5 (2-6)	3 (2-4)	0.5 (0-3)	0.5 (0-3)	3 (1-7)	3 (1-5)	0 (0-0)	0 (0-0)

Abbreviation: DMHT, digital mental health treatment.

Post hoc analyses of the incremental impact of coaching among those with suboptimal engagement found that BCS who received coaching reported significantly reduced depression symptoms at the 8-week postintervention assessment than those who did not receive coaching (mean [SD] PHQ-8 score difference = −4.12 [1.23]; *P* < .001). No significant group differences in depression or anxiety symptoms were observed at the 6-month or 12-month follow-up assessment. Additional details are provided in the eAppendix in Supplement 2.

## Discussion

Overall, the findings from this trial suggest that more sophisticated, interactive designs, such as those in the DMHT program, may confer some incremental benefit compared with simple, less costly, psychoeducational applications such as Mood Education. The DMHT applications appear to offer incremental benefits for addressing mental health needs in BCS, particularly anxiety, supporting the potential of evidence-based DMHTs to help close the mental health treatment gap in this large population.

To our knowledge, this trial was the first to explore the effect of adding human coaching for users who initially had difficulty engaging with a digital intervention. Results suggest that human support may help sustain engagement across days, even if it does not necessarily increase the frequency of application sessions. There were nearly twice as many optimal engagement than suboptimal engagement with DMHT, and while clinical implementation may include a larger proportion of individuals with suboptimal engagement compared with more selected research trials,^55,56^ this finding raises important considerations regarding the cost-effectiveness of providing coaching for all users. Coaching may benefit a subset of BCS, but the relatively small proportion of individuals who appear to require it, the modest improvements in engagement it yields, and its unclear impact on treatment outcomes may not justify the cost of coaching. Nevertheless, our findings suggest that clinicians seeking an adaptive strategy that promotes consistent engagement to a DMHT may consider adding coaching after week 1 for BCS who cannot optimally engage. However, given the limited sample size, findings regarding the benefit of coaching should be interpreted cautiously until replicated in a larger sample.

Both the DMHT and patient education programs led to substantial within-group reductions in depression and anxiety symptoms, raising questions about the types of programs capable of producing meaningful effects. The patient education application, which has also shown benefits in a prior RCT,^52^ provides broad psychoeducational content and was beneficial despite limited sustained engagement, consistent with growing evidence supporting self-guided single-session interventions.^57,58^ As DMHTs increasingly incorporate novel technologies and therapeutic features, more research is needed to determine whether these additions provide incremental benefits over simpler, lower-cost programs in terms of symptom reduction, engagement, and acceptability. Low-intensity interventions may serve as initial stepped-care options, with more intensive treatment reserved for those who need it. Alternatively, simply offering a credible resource may provide reassurance and symptom relief, regardless of engagement level. Future studies including inert control conditions (eg, waiting list, treatment as usual) are needed to clarify these effects.

### Limitations

Limitations of this trial include the lack of diversity in participant race, ethnicity, and educational level, which diminishes the study’s generalizability. Individuals with a college or graduate degree may be more comfortable navigating technology and more likely to complete a rigorous year-long study compared with those with lower educational levels. Due to sample size limitations, comparisons regarding suboptimal engagement of DMHT should be interpreted with caution. Moreover, the absence of a no-treatment or waiting list control makes it impossible to determine whether either program (DMHT or patient education) produces better outcomes than no treatment. While coaches were trained to refrain from providing counseling, it is possible that common elements of psychotherapy (eg, rapport, empathy) occurred in their interactions with participants. Finally, aligned with screening and monitoring methods for distress in cancer care settings, assessments of anxiety and depression symptoms were based on participant self-assessment and not structured clinical interviews.

## Conclusions

In this RCT of a smartphone-delivered DMHT program, the findings indicated that DMHT was efficacious for reducing symptoms of anxiety and, to a lesser extent, depression among BCS with mental health challenges. As the number of BCS continues to increase, so too does the urgency to address growing mental health needs in this population using scalable and highly accessible digital interventions.

## Notes

Supplement 1.
Trial Protocol

Supplement 2.
eAppendix

Supplement 3.
Data Sharing Statement
