# Supplementary material for: Digital Mental Health Treatment and Symptoms of Depression and Anxiety in Breast Cancer Survivors: A Randomized Clinical Trial
Source: JAMA Netw Open. 2026 Jul 20;9(7):e2623871. doi: 10.1001/jamanetworkopen.2026.23871 (PMC13386768; doi:10.1001/jamanetworkopen.2026.23871)
Supplement: Supplement 1. — Trial Protocol [file jamanetwopen-e2623871-s001.pdf]

Note: The following text is from UVA IRB HSR protocol 200218. Only material relevant to the procedures and primary outcome analyses for this study are included. Other aspects of the project that are not relevant to the primary outcome analyses have been removed.

PROTOCOL

Background

1. Provide the scientific background, rationale and relevance of this project.
Breast cancer survivors who experience clinical levels of psychological distress, expressed as symptoms of depression or anxiety, report 2½ times as many unmet psychosocial needs compared to those without significant distress.[1] Fortunately, there are strong data demonstrating that cognitive-behavioral therapy (CBT) is effective at reducing distress of breast cancer survivors.[2–5] However, numerous barriers prevent survivors from receiving services, including financial[6] and time[7] costs, social stigma,[8] and a shortage of therapists.[9–11] Smartphone applications ('apps') hold significant promise to overcome barriers in providing psychosocial care for cancer survivors.[12–15] However, few publicly available apps have been empirically validated for self-management of mood symptoms,[16,17] and those that have been validated have not been tested among cancer survivors.[18] IntelliCare is a suite of apps that utilize a modular skills-based approach for self-managing symptoms of depression and anxiety that has been rigorously studied in the general population,[19–21] but not in cancer populations. Importantly, providing apps alone does not address the widespread problems of suboptimal engagement and gains among most apps,[22,23] including IntelliCare.[20] App-delivered interventions provided with human support generally have better engagement and outcomes,[24,25] yet studies have not tested the efficacy of an adaptive strategy that provides support based on need.

Objectives/Hypothesis

The overarching goals of this project are to provide the first rigorous test of a scalable and publicly accessible mobile health intervention to address distress in women with breast cancer, and to test the impact of human coaching as a way to increase engagement with digital health interventions to improve outcomes.

Aim 1: <u>To test the impact of the IntelliCare apps on symptoms of depression and anxiety in breast cancer survivors</u>. Participants will initially be randomized to receive the IntelliCare apps or app-delivered Patient Education for 8 weeks. We hypothesize that use of the IntelliCare apps will be more efficacious in reducing symptoms of depression and anxiety than app-delivered Patient Education at post-assessment, 6 and 12-month follow-up.

Aim 2: <u>To test the impact of added coaching on engagement with the IntelliCare apps</u>. Participants who receive the IntelliCare apps will be classified as high-engagers or low-engagers based on 1-week app usage patterns. High-engagers will continue to use the apps normally (i.e., no change). The low-engagers will be re-randomized to either receive added coaching vs. not (i.e., no change) in addition to the apps. This will allow

us to evaluate whether added coaching to address barriers to app usage will lead to greater engagement with the apps for individuals that struggle to engage.

Aim 3: To understand breast cancer survivors' perspectives on tailoring the apps and coaching. For this qualitative aim, we will conduct semi-structured exit interviews with participants that receive the IntelliCare apps and coaching. Interviews will capture survivors' perceptions about the extent to which, and how, tailoring the apps and coaching specifically for breast cancer survivors may improve intervention outcomes and engagement.

Study Design: Biomedical

1. **Will controls be used?** Yes.

   ►IF YES, explain the kind of controls to be used. A patient education app with standard information about distress management. This will serve as an active control. All participants will receive something that is expected to benefit them.

2. **What is the study design?**
   The PI and statistician will remain blind to participants' conditions. Given the nature of the interventions being tested, participants cannot be blinded to their condition.

3. **Does the study involve a placebo?** No.

Human Participants

Ages: _18+ years___
Sex: **_female___**
Race: **_any___**

Subjects- see below

1. **Provide target # of subjects (at all sites) needed to complete protocol.** 313

2. **Describe expected rate of screen failure/ dropouts/withdrawals from all sites.**
20%

Inclusion/Exclusion Criteria

1. **List the criteria for inclusion**

(1) age ≥ 18 years;
(2) 0-5 years post-diagnosis of Stage I, II, or III female breast cancer;
(3) elevated symptoms of depression as measured by the Patient Health Questionnaire-8 (PHQ-8) score ≥10 and/or symptoms of anxiety as measured by the Generalized Anxiety Disorder-7 (GAD-7) score ≥8.

2. **List the criteria for exclusion**

(1) Individuals will be permitted to enroll if they are taking medication treatment for depression and/or anxiety and have not had an appointment to adjust the dosage over the past 2 weeks;
(2) mental health condition deemed to interfere with study procedures or put the participant at undue risk based on self-reported history of psychosis or bipolar disorder (see measures), or active suicidal ideation that necessitates more intense care as indicated from the Pitt-Optimum Suicide Safety Protocol the;
(3) do not have an app-compatible phone (i.e., iOS 10.3 or later or Android 4.0.3 or later); and (4) cannot read and speak English (current intervention and coaching only available in English).

Statistical Considerations

1. Is stratification/randomization involved? Yes.

Stage 1 Randomization: After completing the pre-assessment battery, participants will be randomized to 1 of 2 conditions: 1) IntelliCare apps; or 2) App-delivered Patient Education.

Stage 2 Randomization: After monitoring app use for the first week of enrollment, participants who received the IntelliCare apps and are low-engagers of the intervention will be randomly assigned to receive added phone coaching or no change. Those receiving the app-delivered Patient Education (i.e., control condition) will not be re-randomized.

2. What are the statistical considerations for the protocol?
The impact of the IntelliCare apps on mood symptoms will be examined using a potential outcomes framework and marginal structural models (MSM) with appropriate weighting. After data is appropriately weighted, we will use a regression based approach for statistical hypothesis testing. We will use the extended GEE (generalized estimating equation) method for repeated measures models with weights.

3. Provide a justification for the sample size used in this protocol.
The total sample size (N=313) was determined based on the main contrast between the IntelliCare apps and app-delivered Patient Education (Aim 1). A total of 250 breast cancer survivors are needed to detect a small to moderate effect size for symptoms of depression and/or anxiety (Cohen's d) of .35 at 80% power when using a 2-sided, 2-sample t-test with a Type-I error rate of 5%. To account for an estimated study attrition rate of 20% (based on our pilot study), we will recruit a total of 313 breast cancer survivors in order for 250 survivors to complete the study (250÷0.80=313).

4. What is your plan for primary variable analysis?
The impact of the IntelliCare apps on depression and anxiety symptoms will be examined using a potential outcomes framework and marginal structural models (MSM) with appropriate weighting. After data is appropriately weighted, we will use a regression based approach for statistical hypothesis testing. We will use the extended

GEE (generalized estimating equation) method for repeated measures models with weights.

Study Procedures-Biomedical Research

1. What will be done in this protocol?

The apps in this project are collectively known as *IntelliCare*, that were developed originally at Northwestern University and are currently managed and distributed by Adaptive Health, Inc. Each app provides a different type of support. Research personnel will have regular contact with participants throughout the study, so that any questions can be addressed immediately. The coaching manual to be used in this study can be found in the Study Materials folder. The apps are free and are designed for easy and quick use. Participants are free to use the apps as much or as little as they like. The apps are freely available to the public on app stores for both Android and iPhone users. More information can also be found on: https://intellicare.cbits.northwestern.edu/. There are currently 6 apps available for download. Here is a list of *IntelliCare* apps and brief descriptions of what they do:

- **Daily Feats**: Plan and complete activities during the day to increase life satisfaction
- **Worry Knot**: Manage worry with proven worry management techniques
- **Thought Challenger**: Learn how to identify and challenge negative or worrying thoughts
- **Day to Day**: Get tips and tricks to manage your mood
- **My Mantra**: Create positive mantras and construct your own album of encouragement
- **IntelliCare Hub**: Manage to-do's, messages, and reminders of other IntelliCare apps

Participants assigned to the control condition will receive a patient education app that will be freely available to the public on app stores for both Android and iPhone users. It will contain standard content relevant for general distress management. This includes information about thoughts/worries, prevalence of mood symptoms, CBT strategies for coping with negative affect, and other information typical of education-based mental health apps.

The figure (next page) depicts the flowchart of study procedures.

Screening, enrollment, and preassessment: Interested cancer survivors will see our advertisement (Step 1), and respond by either going to our website to read the study description details and/or calling a toll-free number to discuss the details with a member of the research staff. Then, individuals will be directed to complete a brief online interest screen (Step 2) which will be automatically stored and then reviewed by staff to determine initial eligibility (Step 3). Ineligible individuals will be informed that they do not qualify for the study. The study coordinator will speak by phone (Steps 4 and 5) to individuals pre-eligible based on their screener results to inform them about the study, answer all study questions, ensure eligibility, review the consent form, and instruct participants about the online informed consent process. Participants will then

complete the online pre-assessment questionnaire battery online (Step 6). It is estimated that the pre-assessment battery will take 45-60 minutes to complete. For subjects that ultimately sign the consent form, data collected in this screening questionnaire will be de-identified and used in data analysis.

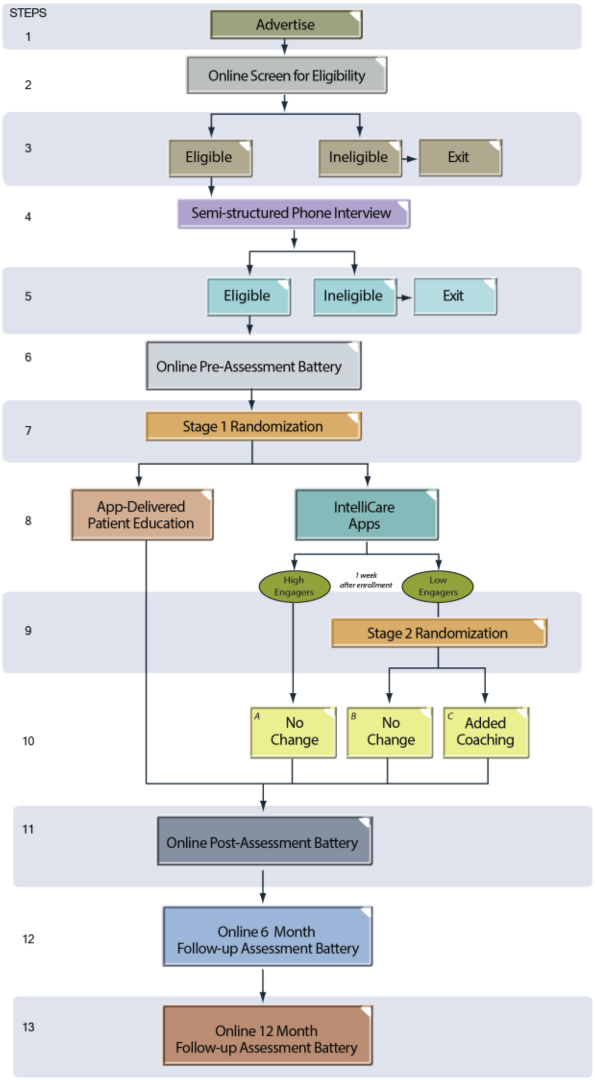

After individuals sign the consent and complete the pre-assessment questionnaire battery, they will be randomly assigned to receive the IntelliCare apps or the patient education app (Step 7). Individuals that receive the patient education app (Step 8) will use it over the 8 weeks without specific instructions about the frequency with which they should access it. The purpose of this is to mimic how individuals typically initiate and use educational mental health apps that are available to the public. Because the IntelliCare apps are free and publicly available, we cannot guarantee that those assigned to the patient education app will not use the IntelliCare apps, though we will strongly discourage them from doing so and will ask them whether they used the IntelliCare apps in the post-assessment questionnaire battery.

For those that are assigned the IntelliCare apps, we will examine their frequency of app use during the first week, based on the Day-to-Day app which is suggested first (Step 8). This will be done through a secure web portal that directly accesses the database from the Amazon cloud where app use is stored by Adaptive Health, which is paid to provide this service. The portal will be password protected to ensure that only study staff can access it. No subject data will be transferred from UVA to Adaptive Health. Based in part on previous data, we will use a cutoff that classifies whether participants are high-engagers or low-engagers of the IntelliCare apps. Participants that are classified as high-engagers will continue to use the apps with no added coaching. Participants who received the IntelliCare apps and are low-engagers of the intervention will be randomly assigned to receive added phone coaching or no change (Step 10). Low-engager individuals who are randomized to the added coaching condition will receive coaching in addition to the standard IntelliCare apps. Coaching will be provided throughout the remainder of the intervention period (until the post-assessment) to those assigned to this condition. We will use the coaching manual provided as part of this protocol, that was developed from the UVA pilot study of the IntelliCare apps among breast cancer survivors at UVA.

Coaching: Coaching is meant to support participants' utilization of the IntelliCare apps. Bachelor's level coaches are part of the study team and target potential points at which users may fail to benefit from the program. These "failure points" include issues related to the usability of the program, engagement with it, fit of the program tool to one's needs, knowledge of how to use the program, and implementation failures (e.g. one practices the skills using the program tools, but does not use them in day to day life). Once a failure point is identified, the coach provides support to the user to help address the obstacles that may be in the way of using the application. Note, coaches will not be providing traditional counseling or psychotherapy. Rather, the coaching is focused on identifying and addressing barriers to application utilization. An initial 30-45 minute coaching call based on the coaching manual will focus on orienting participants to using the apps, setting expectations of the coach's role, assessing how the apps may meet participants' needs, and building rapport. Participants will also be encouraged to contact coaches at any time with any app-related questions. The coaches will be trained and monitored by the PI (Dr. Chow) who has a PhD in clinical psychology and over 8 years of experience in conducting psychological assessments and psychotherapy. Coaches will receive a detailed coaching manual and will attend weekly supervision meetings throughout the trial.

Post-assessment and follow-up assessments: At the end of week 8, regardless of intervention progress or experimental group, participants will be instructed to complete the online post-assessment battery (Step 11). The post-assessment is similar to the pre-assessment battery but also includes the intervention evaluation measures, as used in our previous digital intervention studies, which provide, in part, insight into the utility, preference, and perceived efficacy of the intervention. After completing the post-assessment battery, individuals will have continued access to their assigned program. Those that received added coaching will no longer receive coaching after the start of week nine, but will continue to have access to the apps. This same assessment battery will be completed again at 6 month (Step 12) and 12 month (Step 13) follow-ups.

Bibliography

1. Hodgkinson K, Butow P, Hunt GE, Pendlebury S, Hobbs KM, Wain G. Breast cancer survivors' supportive care needs 2–10 years after diagnosis. *Supportive Care in Cancer*. 2007;15(5):515-523.

2. Tatrow K, Montgomery GH. Cognitive behavioral therapy techniques for distress and pain in breast cancer patients: a meta-analysis. *Journal of Behavioral Medicine*. 2006;29(1):17-27.

3. Gudenkauf LM, Antoni MH, Stagl JM, et al. Brief cognitive–behavioral and relaxation training interventions for breast cancer: A randomized controlled trial. *J Consult Clin Psychol*. 2015;83(4):677-688.

4. Johnson JA, Rash JA, Campbell TS, et al. A systematic review and meta-analysis of randomized controlled trials of cognitive behavior therapy for insomnia (CBT-I) in cancer survivors. *Sleep Med Reviews.* 2016;27:20-28.

5. Zhang M, Huang L, Feng Z, Shao L, Chen L. Effects of cognitive behavioral therapy on quality of life and stress for breast cancer survivors: a meta-analysis. *Minerva Med*. 2017;108(1):84-93.

6. Chi M. The Hidden Cost of Cancer: Helping Clients Cope with Financial Toxicity. *Clinical Social Work Journal*. 2017:1-9.

7. Yabroff KR, Davis WW, Lamont EB, et al. Patient time costs associated with cancer care. *J Natl Cancer Inst*. 2007;99(1):14-23.

8. Holland JC, Kelly BJ, Weinberger MI. Why Psychosocial Care is Difficult to Integrate into Routine Cancer Care: Stigma is the Elephant in the Room. *J Natl Compr Canc Netw*. 2010;8(4):362-366.

9. Davis AS, McIntosh DE, Phelps L, Kehle TJ. Addressing the shortage of school psychologists: A summative overview. *Psychology in the Schools*. 2004;41(4):489-495.

10. Shafran R, Clark D, Fairburn C, et al. Mind the gap: Improving the dissemination of CBT. *Behaviour Research and Therapy*. 2009;47(11):902-909.

11. Kaltenthaler E, Sutcliffe P, Parry G, Beverley C, Rees A, Ferriter M. The acceptability to patients of computerized cognitive behaviour therapy for depression: a systematic review. *Psychological Medicine*. 2008;38(11):1521-1530.

12. Abrol E, Groszmann M, Pitman A, Hough R, Taylor RM, Aref-Adib G. Exploring the digital technology preferences of teenagers and young adults (TYA) with cancer and survivors: a cross-sectional service evaluation questionnaire. *Journal of Cancer Survivorship*. 2017;11(6):670-682.

13. Stubbins R, He T, Yu X, et al. A Behavior-Modification, Clinical-Grade Mobile Application to Improve Breast Cancer Survivors' Accountability and Health Outcomes. *JCO Clinical Cancer Informatics*. 2018;2:1-11.

14. Lubberding S, van Uden-Kraan CF, Te Velde EA, Cuijpers P, Leemans CR, Verdonck-de Leeuw IM. Improving access to supportive cancer care through an e-Health application: a qualitative needs assessment among cancer survivors. *Journal of Clinical Nursing*. 2015;24(9-10):1367-1379.

15. Ringwald J, Marwedel L, Junne F, et al. Demands and needs for psycho-oncological ehealth interventions in women with cancer: cross-sectional study. *JMIR cancer*. 2017;3(2):e19.

16. Bakker D, Kazantzis N, Rickwood D, Rickard N. Mental health smartphone apps: review and evidence-based recommendations for future developments. *JMIR mental health*. 2016;3(1):e7.

17. Donker T, Petrie K, Proudfoot J, Clarke J, Birch M-R, Christensen H. Smartphones for smarter delivery of mental health programs: a systematic review. *J Med Internet Res*. 2013;15(11):e247.

18. Bender JL, Yue RYK, To MJ, Deacken L, Jadad AR. A Lot of Action, But Not in the Right Direction: Systematic Review and Content Analysis of Smartphone Applications for the Prevention, Detection, and Management of Cancer. *J Med Internet Res*. 2013;15(12).

19. Mohr DC, Tomasino KN, Lattie EG, et al. IntelliCare: an eclectic, skills-based app suite for the treatment of depression and anxiety. *J Med Internet Res*. 2017;19(1):e10.

20. Lattie EG, Schueller SM, Sargent E, et al. Uptake and usage of IntelliCare: a publicly available suite of mental health and well-being apps. *Internet Interventions*. 2016;4:152-158.

21. Rubanovich CK, Mohr DC, Schueller SM. Health app use among individuals with symptoms of depression and anxiety: a survey study with thematic coding. *JMIR mental health*. 2017;4(2):e22.

22. Ritterband LM, Thorndike FP, Ingersoll KS, et al. Effect of a web-based cognitive behavior therapy for insomnia intervention with 1-year follow-up: A randomized clinical trial. *JAMA Psychiatry*. 2017;74(1):68-75.

23. Zachariae R, Amidi A, Damholdt MF, et al. Internet-delivered Cognitive-Behavioral Therapy for insomnia in breast cancer survivors: A randomized controlled trial. *J Natl Cancer Inst*. 2018;110(8):880-887.

24. Mohr DC, Cuijpers P, Lehman K. Supportive accountability: A model for providing human support to enhance adherence to eHealth interventions. *J Med Internet Res*. 2011;13(1):e30.

25. Schueller SM, Tomasino KN, Mohr DC. Integrating human support into behavioral intervention technologies: the efficiency model of support. *Clinical Psychology: Science and Practice*. 2017;24(1):27-45.
