# Supplementary material for: Digital Mental Health Treatment and Symptoms of Depression and Anxiety in Breast Cancer Survivors: A Randomized Clinical Trial
Source: JAMA Netw Open. 2026 Jul 20;9(7):e2623871. doi: 10.1001/jamanetworkopen.2026.23871 (PMC13386768; doi:10.1001/jamanetworkopen.2026.23871)
Supplement: Supplement 2. — eAppendix [file jamanetwopen-e2623871-s002.pdf]

Supplemental Online Content

Chow PI, You W, Shaffer KM, et al. Digital mental health treatment and symptoms of depression and anxiety in breast cancer survivors: a randomized clinical trial. *JAMA Netw Open*. 2026;9(7):e2623871. doi:10.1001/jamanetworkopen.2026.23871

eAppendix

This supplemental material has been provided by the authors to give readers additional information about their work.

© 2026 Chow PI et al. *JAMA Network Open.*

eAppendix

Because the Missing At Random (MAR) assumption underlying weighted GEE cannot be empirically tested, we conducted sensitivity analyses to evaluate the robustness of the primary findings. Specifically, we performed a complete-case analysis restricted to participations with outcomes observed at all follow-up assessments. Results were similar in direction and magnitude to those of the primary analysis.

We further compared the two embedded dynamic treatment regimens (EDTRs) among participants randomized to IntelliCare at Stage 1: (1) IntelliCare without added coaching, in which all participants continued IntelliCare regardless of early engagement, and (2) IntelliCare with coaching augmentation, in which all participants were suboptimal engagers and received added coaching. Population-averaged EDTR effects were estimated using inverse probability-weighted GEE, with weights reflecting the SMART randomization probabilities and robust standard errors. A statistically significant group difference in depression symptoms was observed at the post-intervention assessment, with lower symptoms among participants in the IntelliCare-with-Coaching EDTR compared with those in IntelliCare-without-Coaching EDTR (mean difference of PHQ score = -1.46, SD=0.59, p=0.01). No statistically significant group differences were observed at the 6- or 12-month follow-up assessments. No significant group differences were observed for anxiety symptoms at any assessment timepoints.

As exploratory analyses, we compared second-stage intervention options among IntelliCare participants who demonstrated suboptimal engagement during the first week and were therefore eligible for re-randomization. Specifically, suboptimal IntelliCare engagers who received coaching (n=34) were compared with those who did not receive coaching (n=38) at each post-baseline assessment relative to baseline. A statistically significant group difference in depression symptoms was observed at the post-intervention assessment, with lower symptoms among participants who received added coaching compared with those who did not (mean difference of PHQ score = -4.12, SD=1.23 $p<.001$). No

© 2026 Chow PI et al. *JAMA Network Open.*

statistically significant group differences were observed at the 6- or 12-month follow-up assessments. No significant group differences were observed for anxiety symptoms at any assessment timepoints.

© 2026 Chow PI et al. *JAMA Network Open*.
