# Supplementary material for: Digital Mental Health Treatment and Symptoms of Depression and Anxiety in Breast Cancer Survivors: A Randomized Clinical Trial
Source: JAMA Netw Open. 2026 Jul 20;9(7):e2623871. doi: 10.1001/jamanetworkopen.2026.23871 (PMC13386768; doi:10.1001/jamanetworkopen.2026.23871)
Supplement: Supplement 3. — Data Sharing Statement [file jamanetwopen-e2623871-s003.pdf]

Data Sharing Statement

Chow. Digital Mental Health Treatment and Symptoms of Depression and Anxiety in Breast Cancer Survivors. *JAMA Netw Open*. Published July 20, 2026.
doi:10.1001/jamanetworkopen.2026.23871

Data
Additional Information: clinicaltrials.gov, NCT04583891
Data available: Yes
Data types: Deidentified participant data
How to access data: pic2u@virginia.edu
When available: With publication

Supporting Documents
Document types: None

Additional Information
Who can access the data: Qualified researchers with appropriate expertise conducting rigorous, independent, and novel scientific research.
Types of analyses: For a specified purpose.
Mechanisms of data availability: De-identified row-level data may be available upon reasonable request to the corresponding author and a data use agreement with the University of Virginia per institutional requirement.
